# Supplementary material for: Nutritional Quality of Pre-Packaged Foods in China under Various Nutrient Profile Models
Source: Nutrients. 2022 Jun 29;14(13):2700. doi: 10.3390/nu14132700 (PMC9268697; doi:10.3390/nu14132700)
Supplement: Supplementary file 1 [file nutrients-14-02700-s001.zip › nutrients-1753435-supplementary.pdf]

Nutritional quality of pre-packaged foods in China under various nutrient profile models

Supplement file

Contents

Table S1. Description of the fourteen food groups 2

Table S2. Definition of added sugar, salt, fat, non-sugar sweetener and food additive in ingredients 6

Table S3. Criteria of the PAHO NPM, Chilean NPM and China NRV 7

Table S4. WHO nutrient profile model for the Western Pacific region (WPHO NPM) 8

Table S5. Proportion of pre-packaged foods exceeding the Chilean NPM criteria 10

Table S6. Proportion of pre-packaged foods exceeding the PAHO NPM criteria 11

Table S7. Proportion of pre-packaged foods exceeding the WPHO NPM criteria 12

Table S1. Description of the fourteen food groups

Food group	Food category	Description
**Beverages**	Juices	Fresh and ambient fruit and vegetable juices
	Soft drinks	Sugar-sweetened and artificially sweetened soft drinks
	Electrolyte drinks	Sports electrolyte drinks
	Waters	Plain and flavored waters
	Coffee and tea	All coffee and tea products
	Powdered beverages	All beverage mixes
**Bread and bakery products**	Bread	White, wholemeal and mixed grain/seed sliced bread and rolls
		Fruit bread and fruit-based rolls
		Wraps and other flatbread products
		Turkish pide, bagels, English-style muffins, crumpets, pizza bases and other plain bread-based products
	Biscuits and cookies	Filled and unfilled sweet biscuits
		Flavored and plain crisp bread and crackers
	Cakes, muffins and pastry	Scones, pikelets, doughnuts, cakes, sweet buns, pancakes, crepes, muffins (cake-style), slices etc
		Cake, pikelet and pancake dry mixes
		Sweet pastries (fresh, ambient, chilled and frozen)
**Cereal and grain products**	Breakfast cereal	Ready to eat breakfast cereals
		Oats and other breakfast cereals that require heating
		Other processed cereals (e.g. bran)
	Cereal and nut-based bars	Plain, chocolate-topped and yoghurt-topped cereal-based bars
	Noodles	Plain dry noodles
		Savory/flavored dry noodle-based dishes
	Pasta	Canned and ambient pasta and sauce (with and without meat) products (excludes frozen ready meals)
		Packaged fresh pasta with sauce
		Savory/flavored dry pasta-based side dishes

		Plain dry pasta
	Rice	Plain rice
		Savory rice-based side dishes
	Other breakfast products	All breakfast products not included in "Breakfast cereal"
	Other cereal products	Flour and other cereals (e.g. polenta, cous cous, bread crumbs, yeast)
**Confectionery**	Chocolate and sweets	Chocolate-based confectionery, sugar-based confectionery
	Chewing gum	All sugar-sweetened and sugar-free chewing gums and bubble gum products
**Convenience foods**	Pizza	Frozen and refrigerated pre-packed pizzas
	Soup	Canned, chilled and ambient soup products
	Ready meals	Frozen, chilled and ambient pre-prepared meals
	Meal kits	Kits with ingredients to put meals together
	Pre-prepared salads and sandwiches	Chilled pre-prepared salads and sandwiches
	Other convenience foods	Other pre-prepared foods not included in above categories
**Dairy and substitutes**	Cheese	Feta, haloumi, parmesan and other high-salt cheeses
		All types of full and reduced fat cheddar/Colby etc cheese including shredded, block or sliced
		Soft cheeses such as cream cheese, ricotta and cottage cheese
		Processed cheese slices and products
	Cream	Thickened, sour and regular cream products
	Dairy desserts	Dairy-based desserts (e.g. custards, rice puddings)
		Dairy-based dessert mixes (e.g. powders)
	Ice cream and edible ices	Dairy and non dairy-based ice cream varieties and edible ices
	Milk products	Flavored and unflavored dairy milk products
		Flavored and unflavored soymilks
		Flavored and unflavored oat, almond and other milks
		Condensed, evaporated and powdered milk products (including coconut milk)
	Yoghurt products	Fruit, flavored, natural and plant-based yoghurts (full fat, reduced fat and skim varieties) including yoghurt drinks

**Edible oils**		Salted and unsalted butter and margarine products, vegetable oils
**Egg and egg products**		Fresh, sauced, preserved and salted eggs
**Fruit, vegetables, nuts and legumes**	Fruit	Dried fruit products including coconut
		Fruit-based bars
		Fruit products canned in juice or syrup
		Fruit gels, fruits in jelly and fruit puree
	Jam and fruit spreads	Jams, marmalades and other preserves
	Nuts and seeds	Salted and unsalted nuts and seeds
	Vegetables	Canned tomato products
		Canned beans and peas
		Baked beans in tomato sauce (with and without additions)
		Canned creamed, plain and sweet corn
		All other canned vegetables
		Pickled vegetable and olive products
		Frozen potato-based products
		Frozen vegetables
**Meat and meat alternatives**	Meat alternatives	Plain tofu and other meat-free alternatives
		Meat-free products (e.g. meat-free sausages)
	Processed meat	Pre-packed bacon products
		Beef, pork, chicken and lamb sausages and chilled hot dogs
		Pre-packaged sliced deli meats
		Pre-packaged salami and cured meats
		Beef, pork, chicken and lamb meat burgers
		Canned meat products (excluding soup and pasta)
		Frozen meat pies, sausage rolls and other meat-based pastry products
**Sauces, dressings and condiments**	Mayonnaise and salad dressings	Full and low-fat mayonnaise
		Oil-based, vinegar-based and other types of salad dressing
	Sauces	Table sauces such as tomato sauces and ketchups, sweet chilli, BBQ sauces
		Steak, HP and Worcestershire sauces

		Soy, fish, oyster and other Asian high-salt sauces
		Mustard products
		Marinade products
		Meat accompaniments (e.g. apple, cranberry and mint sauces)
		Plain and flavored tomato paste products
		Asian and Indian flavored powdered, ambient and liquid meal-based sauces
		Ambient and fresh pasta sauces
		Recipe bases
		Liquid and powdered gravies and stock
	Spreads	Crunchy and smooth salted and unsalted peanut butter
		Relishes, chutneys and pickles
		Other savory spreads (e.g. vegetable spreads)
		Pâté spreads
		Sweet spreads
		Yeast-extract spreads (e.g. vegemite)
		Chilled and ambient dips and salsa
**Fish and fish products**	Canned seafood	All varieties of plain and flavored canned seafood
	Chilled and frozen seafood	Chilled processed fish and other seafood products (e.g. smoked salmon)
		Coated frozen fish products (e.g. fish fingers) and uncoated fish products
**Snack foods**		Plain and flavored potato crisps
		Plain and flavored snack foods
		Extruded snacks (e.g. cheesy snacks)
		Plain and flavored corn chips
		Pretzels, popcorn and other snack foods
		Other fried snack foods (e.g. plantain chips)
		All varieties of cracker-based snack packs
**Sugars, honey and related products**	Honey and syrups	Honey, golden, maple and other syrups
		Dessert toppings
		Sugar and artificial sweeteners

Table S2. Definition of added sugar, salt, fat, non-sugar sweetener and food additive in ingredients

**Definition**	**Keywords in ingredients**
Added sugar[1]	sugar, honey, syrup, molasses, maltodextrin, glucose, fructose, concentrated juice, chocolate, milk sugar, trehalose, condensed milk, red sugar, black sugar, lactose, maltdose, corn syrup, high fructose corn syrup, maple syrup, sucrose, refined cane sugar
Added salt[1]	salt, sodium chloride, cheese, processed meat, sauce
Added fat[1]	oil, butter, cream, fat, vegetable oil
Added non-sugar sweetener[2]	neotame, ammonium glycyrrhizinate, monopotassium and tripotassium glycyrrhizinate, D-mannitol, sodiumcyclamate, calciumcyclamate, maltitol and maltitol syrup, lactitol, sucralose, sorbitol and sorbitol syrup, thaumatin, sodiumsaccharin, alitame, aspartame, aspartame-acesulfame salt, steviolglycosides, acesulfame potassium, isomaltulose (palatinose), erythritol, lo-han-kuo extract, xylitol, polydextrose and other sugar alcohols
Added food additives beyond sweeteners	259 food additives of 21 categories listed in National food safety standard for use of food additives (GB 2760-2014). Here listed the 21 categories:<br>acidity regulator, anti caking agent, defoamer, antioxidant, bleaching agent, bulking agent, basic ingredients in gum based candy, colorant, color fixative, emulsifier,<br>enzyme preparation, flavour enhancer, flour treatment agent, coating agent, water retention agent, preservative, stabilizer and coagulators, thickening agent, fragrance used in food, food processing aids and others.

Note: Foods containing any one of the keywords in ingredients in Chinese with either scientific name or trade name were defined as adding the corresponding nutrients or food additives.

[1]The definition of added sugar, salt, fat were in reference to *Public Health Nutr* 2021;24:1514-25.doi: 10.1017/S1368980019005056 and adapted by researchers according to the actual ingredients of pre-packaged foods in China.

[2]The first 19 keywords of sweeteners are permitted by Food Additive Standard of China (GB2760-2014) http://www.nhc.gov.cn/sps/s3593/201412/d9a9f04bc35f42ecac0600e0360f8c89.shtml (accessed on 25 March 2022), and two other sweeteners including polydextrose and other sugar alcohols such as fruit glucitol and multiple sugar alcohol are also included to reflect the actual ingredients of pre-packaged foods in China.

Table S3. Criteria of the PAHO NPM, Chilean NPM and China NRV

Nutrients	PAHO NPM	Chilean NPM in 2019	China NRV
Total fat	≥30% of total energy	-	≤60g & ≤ 30% of total energy
Saturated fat	≥10% of total energy	Solids: ≥4 g/100 g<br>Liquids: ≥3 g/100mL	< 10% of total energy
Trans fat	≥ 1% of total energy	-	≤ 2.2 g/d & < 1% total energy
Sugar	≥ 10% of total energy from free sugars	Solids: ≥10 g/100 g<br>Liquids: ≥5 g/100 mL	≤ 10% of total energy & ≤ 50 g/d of added sugars *
Sodium	≥ 1 mg/4.2 kJ (1kcal)	Solids: ≥400 mg/100 g<br>Liquids: ≥100 mg/100 mL	≤ 2000 mg/d & salt ≤ 6g/d
Energy	-	Solids: ≥1150 kJ (275 kcal) /100 g<br>Liquids: ≥233 kJ (70 kcal) /100 mL	≤ 8400kJ/d
Non-sugar sweeteners	Presence	-	-
Eligibility for being rated	Processed and ultra-processed foods according to NOVA classification	Foods with added saturated fat, sugar, and sodium	All foods

* The reference value of added sugar came from the WHO sugar guideline (≤ 10% of total energy) and the dietary guidelines for Chinese residents (≤ 50 g/d of added sugars).

Table S4. WHO nutrient profile model for the Western Pacific region (WPHO NPM)

Item	Food category	Marketing prohibited if exceeds per 100 g						
		Total fat (g)	Saturated fat (g)	Total sugars (g)	Added sugars (g)	Non-sugar sweetener	Sodium (g)	Energy (kcal)
1	Chocolate and sugar confectionery, energy bars, and sweet toppings and desserts	Not permitted						
2	Cakes, sweet biscuits and pastries, other sweet bakery products, dry mixes for making such	Not permitted						
3	Savory snacks				0		0.04	
4	Beverages<br>a) Juices			5		0		
	b) Milk drinks	4			0	0		
	c) Energy drinks, tea and coffee	Not permitted						
	d) other beverages				0	0		
5	Edible ices	4		10		0	0.08	
6	Breakfast cereals	10		15		0	0.64	
7	Yogurts, sour milk, cream, other similar foods	4		10		0	0.08	
8	Cheese	20					0.52	
9	Ready-made and convenience foods and composite dishes	10	4	10			0.4	225

10	Butter and other fats and oils		20		0	0	0.56	
11	Bread, bread products<br>and crisp breads	10		10			0.48	
12	Fresh or dried noodles, pasta, Dried and fresh noodles, sago, tapioca<br>rice and grains	10		10			0.48	
13	Fresh and frozen meat, poultry, fish and similar	20						
14	Processed meat, poultry,<br>fish and similar	20					0.68	
15	Fresh and frozen fruit, vegetables and legumes	20					0.68	
16	Processed fruit, vegetables<br>and legumes	5		10	0		0.4	
17	Products made from soya	12		10	0		0.4	
18	Sauces, dips and dressings	10			0		0.4	

Note: Cited from WHO nutrient profile model for the Western Pacific Region: a tool to protect children from food marketing. World Health Organization 2016.

Table S5. Proportion of pre-packaged foods exceeding the Chilean NPM criteria

Category	N1*	Excessive n (%)	Energy n (%)	Sodium n (%)	N2*	Saturated fat n (%)	N3*	Total sugar n (%)
Food groups								
Bread and bakery products	10355	10057 (97.1)	10017 (96.7)	1800 (17.4)	639	484 (75.7)	616	354 (57.5)
Cereal and cereal products	3077	2779 (90.3)	2666 (86.6)	1256 (40.8)	175	30 (17.1)	164	98 (59.8)
Confectionary	5313	4420 (83.2)	4380 (82.4)	98 (1.8)	331	249 (75.2)	539	195 (36.2)
Convenience food	3128	2467 (78.9)	1741 (55.7)	2241 (71.6)	81	51 (63.0)	62	3 (4.8)
Dairy products	4856	2842 (58.5)	2729 (56.2)	332 (6.8)	188	90 (47.9)	263	132 (50.2)
Egg and egg products	283	260 (91.9)	14 (4.9)	257 (90.8)	0		0	
Fish and fish products	1049	888 (84.7)	347 (33.1)	866 (82.6)	32	6 (18.8)	34	0(0)
Fruits and vegetables	10319	8450 (81.9)	6321 (61.3)	4380 (42.4)	193	20 (10.4)	200	95 (47.5)
Meat and meat products	5126	4885 (95.3)	1980 (38.6)	4843 (94.5)	41	13 (31.7)	41	20 (48.8)
Non-alcoholic beverages	5695	4506 (79.1)	1752 (30.8)	160 (2.8)	324	34 (10.5)	5693	4439 (78.0)
Snack foods	6224	6087 (97.8)	5723 (92.0)	4611 (74.1)	388	279 (71.9)	431	55 (12.8)
Foods or Beverages								
Beverages	7501	5490 (73.2)	2795 (37.3)	156 (2.1)	411	60 (14.6)	4905	3661 (74.6)
Foods	47924	42151 (88.0)	34875 (72.8)	20688 (43.2)	1981	1196 (60.4)	3138	1730 (55.1)
NOVA groups								
Processed foods	8422	7193 (85.4)	5993 (71.2)	2927 (34.8)	346	99 (28.6)	1012	770 (76.1)
Ultra-processed foods	47003	40448 (86.1)	31677 (67.4)	17917 (38.1)	2046	1157 (56.5)	7031	4621 (65.7)
Total	55425	47641 (86.0)	37670 (68.0)	20844 (37.6)	2392	1256 (52.5)	8043	5391 (67.0)

* N1: Number of excessive and products for energy and sodium; N2: Number of products for saturated fat; N3: Number of products for total sugar.
Percentage of saturated fat and total sugar was based on non-missing values. Excessive = any one of the nutrients exceeded the criteria. - = no data was reported.

Table S6. Proportion of pre-packaged foods exceeding the PAHO NPM criteria

Category	N1*	Excessive n (%)	Fat n (%)	Sodium n (%)	NSS n (%)	N2*	Saturated fat n (%)	N3*	Trans fat n (%)	N4*	Free sugar n (%)
Food groups											
Bread and bakery products	10355	8852 (85.5)	7871 (76.0)	1385 (13.4)	2253 (21.8)	639	461 (72.1)	10355	124 (1.2)	616	343 (55.7)
Cereal and cereal products	3077	1856 (60.3)	492 (16.0)	1335 (43.4)	315 (10.2)	175	28 (16.0)	3077	4 (0.1)	164	96 (58.5)
Confectionary	5313	3163 (59.5)	1648 (31.0)	292 (5.5)	1387 (26.1)	331	242 (73.1)	5313	49 (0.9)	539	193 (35.8)
Convenience food	3128	2790 (89.2)	1819 (58.2)	2584 (82.6)	593 (19.0)	81	57 (70.4)	3128	5 (0.2)	62	18 (29.0)
Dairy products	4856	3939 (81.1)	2766 (57.0)	1293 (26.6)	1721 (35.4)	188	150 (79.8)	4856	19 (0.4)	263	179 (68.1)
Egg and egg products	283	281 (99.3)	262 (92.6)	276 (97.5)	2 (0.7)	0		283	0(0)	0	
Fish and fish products	1049	1036 (98.8)	650 (62.0)	1001 (95.4)	48 (4.6)	32	20 (62.5)	1049	4 (0.4)	34	14 (41.2)
Fruits and vegetables	10319	7578 (73.4)	4488 (43.5)	4334 (42.0)	3577 (34.7)	193	34 (17.6)	10319	4 (0.0)	200	128 (64.0)
Meat and meat products	5126	5092 (99.3)	3259 (63.6)	5023 (98.0)	160 (3.1)	41	24 (58.5)	5126	8 (0.2)	41	20 (48.8)
Non-alcoholic beverages	5695	5612 (98.5)	554 (9.7)	1492 (26.2)	1965 (34.5)	324	48 (14.8)	5695	8 (0.1)	5693	5155 (90.5)
Snack foods	6224	5944 (95.5)	4772 (76.7)	4153 (66.7)	2058 (33.1)	388	163 (42.0)	6224	14 (0.2)	431	45 (10.4)
Foods or Beverages											
Beverages	7501	7016 (93.5)	1587 (21.2)	2165 (28.9)	3151 (42.0)	411	89 (21.7)	7501	0(0)	4905	4365 (89.0)
Foods	47924	39127 (81.6)	26994 (56.3)	21003 (43.8)	10928 (22.8)	1981	1138 (57.4)	47924	239 (0.5)	3138	1826 (58.2)
NOVA groups											
Processed foods	8422	6220 (73.9)	3900 (46.3)	3117 (37.0)	0(0)	346	118 (34.1)	8422	19 (0.2)	1012	834 (82.4)
Ultra-processed foods	47003	39923 (84.9)	24681 (52.5)	20051 (42.7)	14079 (30.0)	2046	1109 (54.2)	47003	220 (0.5)	7031	5357 (76.2)
Total	55425	46143 (83.3)	28581 (51.6)	23168 (41.8)	14079 (25.4)	2392	1227 (51.3)	55425	239 (0.4)	8043	6191 (77.0)

* N1: Number of excessive and products for fat, sodium and NSS; N2: Number of products for saturated fat; N3: Number of products for trans fat; N4: Number of products for free sugar. Percentage of saturated fat and total sugar was based on non-missing values. Excessive = any one of the nutrients exceeded the criteria. NSS=Non-sugar sweeteners. - = no data was reported.

Table S7. Proportion of pre-packaged foods exceeding the WPHO NPM criteria

Category	N1*	Excessive n (%)	N2*	Energy n (%)	Fat n (%)	Sodium n (%)	Added sugar n (%)	NSS n (%)	N3*	Saturated fat n (%)	N4*	Total sugar n (%)
Food groups												
Bread and bakery products	10355	9750 (94.2)	1242	0(0)	613 (49.4)	43 (3.5)	0(0)	0(0)	16	0(0)	15	7 (46.7)
Cereal and cereal products	3077	1824 (59.3)	3069	2 (0.1)	652 (21.2)	1137 (37.0)	0(0)	160 (5.2)	175	0(0)	164	86 (52.4)
Confectionary	5313	5313 (100.0)										
Convenience food	3128	2666 (85.2)	3128	2103 (67.2)	1599 (51.1)	2237 (71.5)	0(0)	0(0)	81	51 (63.0)	62	3 (4.8)
Dairy products	4856	3942 (81.2)	4734	0(0)	1899 (40.1)	659 (13.9)	1530 (32.3)	1669 (35.3)	187	0(0)	261	97 (37.2)
Egg and egg products	283	187 (66.1)	283	0(0)	10 (3.5)	184 (65.0)	0(0)	0(0)	0		0	
Fish and fish products	1049	602 (57.4)	1049	0(0)	229 (21.8)	541 (51.6)	0(0)	0(0)	32	0(0)	34	0(0)
Fruits and vegetables	10319	9863 (95.6)	10319	0(0)	1495 (14.5)	5715 (55.4)	8232 (79.8)	30 (0.3)	193	0(0)	200	70 (35.0)
Meat and meat products	5126	4314 (84.2)	5126	0(0)	698 (13.6)	4228 (82.5)	0(0)	0(0)	41	0(0)	41	0(0)
Non-alcoholic beverages	5695	5557 (97.6)	4606	0(0)	0(0)	0(0)	3337 (72.4)	1834 (39.8)	267	0(0)	4604	864 (18.8)
Snack foods	6224	6207 (99.7)	6224	0(0)	0(0)	6005 (96.5)	5612 (90.2)	0(0)	388	0(0)	431	0(0)
Foods or Beverages												
Beverages	7501	7181 (95.7)	7066	0(0)	959 (13.6)	324 (4.6)	4127 (58.4)	3066 (43.4)	391	0(0)	4470	918 (20.5)
Foods	47924	43044 (89.8)	32714	2105 (6.4)	6236 (19.1)	20425 (62.4)	14584 (44.6)	627 (1.9)	989	51 (5.2)	1342	209 (15.6)
NOVA groups												
Processed foods	8422	6767 (80.3)	7491	146 (1.9)	1135 (15.2)	3537 (47.2)	2678 (35.7)	0(0)	263	1 (0.4)	757	346 (45.7)
Ultra-processed foods	47003	43458 (92.5)	32289	1959 (6.1)	6060 (18.8)	17212 (53.3)	16033 (49.7)	3693 (11.4)	1117	50 (4.5)	5055	781 (15.5)
Total	55425	50225 (90.6)	39780	2105 (5.3)	7195 (18.1)	20749 (52.2)	18711 (47.0)	3693 (9.3)	1380	51 (3.7)	5812	1127 (19.4)

* N1: Number of excessive; Number of
products for energy, fat, sodium, added sugar and NSS; N3: Number of products for saturated fat; N4: Number of products for free sugar.

Three food groups (Chocolate and sugar confectionery, energy bars, and sweet toppings and desserts; Cakes, sweet biscuits and pastries, other sweet bakery products, dry mixes for making such; Energy drinks, tea and coffee) that were not permitted for marketing in the WPHO NPM were defined as excessive in total but were excluded from the analysis of individual nutrient.

Percentages of saturated fat and total sugar were based on non-missing values. Added sugar was based on whether it was contained in ingredients. Excessive = any one of the nutrients exceeded the criteria. NSS = Non-sugar sweeteners. - = no data was reported.
